# Supplementary material for: Long-Term Assessment of Captive Chimpanzees: Influence of Social Group Composition, Seasonality and Biographic Background
Source: Animals (Basel). 2023 Jan 26;13(3):424. doi: 10.3390/ani13030424 (PMC9913678; doi:10.3390/ani13030424)
Supplement: Supplementary file 1 [file animals-13-00424-s001.zip › animals-2163747-supplementary.pdf]

Supplementary Material: Long-term assessment of captive chimpanzees: Influence of social group composition, seasonality and biographic background

Arnau Pascual[1,2], Elfriede Kalcher-Sommersguter[3], David Riba[1,4], Dietmar Crailsheim[1,5]

Table S1. Chimpanzee Behavior Ethogram

**Abnormal**	This includes: (a) Atypical Behaviors, i.e. behaviors not typically seen in wild populations; (b) behaviors exhibited in an excessive manner or as an inadequate, non-adaptive response to a specific situation. SELF SCRATCH: invariable, rhythmic and repetitive movement of the hand fingers or with an object (e.g. stick) rubbing some part of its own body. SELF POKE: insert or press fingers or objects (e.g. sticks) repeatedly or continuously in a part of the body (only mark when actively manipulating or maintaining contact). SELF CLAP: hitting (soft or hard) some part of the body with hands or feet rhythmically and repetitively. ROCKING: Repetitive and invariable swinging of the body while sitting (they can hug or grab objects at the same time, e.g. pile of straw). OVER GROOMING: excessive self-cleaning behavior of the skin / coat with hands or mouth, which may include pulling and ingesting hair. The behavior can also be focused on wounds or damage to the skin, deteriorating its condition. They usually do it on the same areas of the body with a visible lack of hair. COPROPHAGY: handling and/or ingestion of feces (both own and others).
**Locomotion**	Displacement from a point A to a point B on a vertical or horizontal surface, while not doing any other behavior from the catalogue.
**Alimentation**	All behaviors related to food. FOOD: ingest or handle solid or liquid food in a stationary position. FOODING: active search and/or transport of food (the animal keeps its eyes focused on possible food sources). It can include punctual food intake without interrupting gathering food. FOOD EXTRACTION: The chimpanzee is working an enrichment device/item (provided by care givers) which contains food items. This may include longer periods of manipulation in order to bring out the food items which are occasionally ingested. The chimpanzee might use feet, hands, mouth or tools.
**Manipulation**	Using and/or inspecting elements of the environment or enrichment that are not food with the upper or lower extremities.
**Inactivity**	RESTING: the individual remains stationary without actively interacting with himself, the environment or other individuals, nor exhibits behaviors explained in VIGILANCE or RELAXED behaviors. VIGILANCE BEHAVIOR: described as the fixed gazing of something concrete or scanning the distant environment in a fixed, immobile position, either seated or bipedal standing. It does not include looking at parts of your own body, objects in close proximity, or other individuals within a 5m radius. It includes looking at distant group members, other groups, and the surrounding area. RELAXED RESTING: dozing or sleeping in an upward-facing or lateral position with either elongated or bent limbs (without hugging oneself). The individual does not show any indication of being focused on any other individual or their general environment.
**Self-Directed**	Behaviors directed towards the individual, such as self-cleaning (auto grooming), masturbation, scratching, scrubbing, body inspection, etc.
**Other Individual**	Individual behaviors that are not better defined by any other category of the "Solitary Behavior" group.

**Solitary Play**	All Solitary Play behaviors have to be accompanied by either laughs or a "play face". Whimsical behavior that does not include any other conspecific.
**Human Interaction**	Interaction, or looking for interaction, with humans of a neutral type, or without showing clear signs of antagonism. HUMAN AFFILIATIVE INTERACTION: search, action, or mutual influence with or without physical contact between animal and human of non-agonistic type. It can be initiated by animals or by humans. OBSERVING HUMAN CLOSE UP: the individual remains less than 1.5 meters from the fence observing humans in close proximity. FOLLOW HUMAN: locomotion in parallel to the fence following the path that people make around the enclosure. HUMAN AGGRESSION: chimpanzees show threatening behavior and/or perform an agonistic display directed at humans in close proximity. It may include attempts to come into physical contact, throw items, or spit. HUMAN SUBMISSIVE: Chimpanzees are agitated with a scared face can include behaviors such as asking for support, crying or yelling directed at a human.
**Grooming**	Body-cleansing behavior (grooming) from one individual to another (includes mutual grooming), performed with the upper extremities or with the mouth.
**Agonistic Dominance**	Behaviors relating to the agonistic/display threat, direct aggression, displacement and resource appropriation (including object and social resources) It can be accompanied by vocalizations.
**Agonistic Submission**	The chimpanzee shows signs of fear directed at another individual in an aggressive context. It may include approaching and presenting vulnerable body parts in an attempt to appease or attempt to avoid or flee from an individual displaying aggressive behavior. It can include actions such as genital presenting, extending limbs, hand to mouth, finger to mouth, running away, avoiding, and is typically accompanied by a scared face and vocalizations such as yelling, screaming, crying or pant-grunting.
**Other Agonistic**	Other aggression-related behaviors that cannot be defined by the Agonistic Dominance or Agonistic Submission categories.
**Social Play**	Playful behavior between two or more individuals punctually accompanied by play indicators (e.g., play face, laugh, gallop, cartwheels and pirouettes). Tag, catch and grab limbs or objects, tickles, muzzling, rough-and-tumble. These interactions do not require permanent physical contact.
**Other Affiliative**	Other affiliative-like behaviors that cannot be defined by the “Social Play” or “Grooming” categories. Any of the following 3 subcategories are marked if they fit the following definitions: FOLLOW: Synchronized movement following the trajectory of another individual locomotion following the trajectory of another individual, moving beside or behind (always mark mutual). EMBRACE: An individual hugs or tries to hug another with one or both arms. FEED TOGETHER Two individuals eat from the same food source or forage in close proximity allowing the collection of food within their reach by another individual, sharing the food in a passive way (always marking it mutual).
**Socio Sexual**	Sexual interaction, or search for sexual interaction, between two individuals that includes behaviors such as: copulation, attempted copulation, genital presentation, and other behaviors directed towards the genitals of another individual.
**Not Visible**	The individual cannot be located or is out of the view of the observer.

Table S2. DEWD Full-model.

**DEWD Full-model**			
	Estimate	Std. Error	t value
(Intercept)	0.18780	0.11216	1.674
Group size	-0.03532	0.05576	-0.633
Sex ratio	-0.04757	0.06429	-0.740
Seasonality	0.02962	0.04500	0.658
Sex	0.05848	0.07368	0.794
Origin	0.08760	0.07002	1.251

Table S3. VSC Full-model.

**VSC Full-model: Δ AIC = 5.85; p = 0.007301 ****					
	Estimate	Std. Error	df	t value	Pr(>|t|)
(Intercept)	5.8599	0.9690	15.6207	6.047	1.88e-05 ***
Sex	-2.5125	0.8469	8.0560	-2.967	0.01782 *
Seasonality	-0.7708	0.2743	27.426	-2.810	0.00907 **
Sex ratio	-0.5473	0.3978	27.7722	-1.376	0.17990
Group size	-0.4061	0.3446	27.7328	-1.178	0.24870
Origin	-2.5801	0.8344	7.6113	-3.092	0.01578 *

Table S4. Results from the post hoc tests on the VSC Full-model.

**Type III Analysis of Variance Table with Satterthwaite's method**							**Pairwise comparison of means (emmeans)**						
	Sum Sq	Mean Sq	NumDF	DenDF	F value	Pr(>F)	**Fixed Factor**	**Estimate**	**Std. Error**	**df**	**t ratio**	**p-value**	**Cohen's *d***
Sex	5.6291	5.629	1	8.0560	8.8016	0.017821*	F - M	2.51	1.1	12.9	2.281	0.0401 *	0.8816
Seasonality	5.0496	5.0496	1	27.2426	7.8955	0.009065**	Cold - Warm	0.771	0.291	30.6	2.645	0.0128 *	0.4403
Sex ratio	1.2104	1.2104	1	27.7722	1.8926	0.179904	-	-	-	-	-	-	
Group size	0.8879	0.8879	1	27.7328	1.3883	0.248699	-	-	-	-	-	-	
Origin	6.1151	6.1151	1	7.6113	9.5616	0.015776*	Captive - Wild	2.58	1.09	12.2	2.366	0.0353 *	1.2014

Signif. codes: '***' ≤ 0.001 '**' ≤ 0.01 '*' ≤ 0.05 '.' ≤ 0.1 ' ' ≤ 1

Table S5. General Activity Index Full-model.

General Activity Index Full-model: Δ AIC = 31.5; p = 7.276e-08 ***					
	Estimate	Std. Error	df	t value	Pr(>|t|)
(Intercept)	0.53866	0.09114	25.05128	5.910	3.59e-06 ***
Group size	-0.15210	0.04056	28.69266	-3.750	0.000795 ***
Sex ratio	-0.23554	0.04679	28.77319	-5.034	2.36e-05 ***
Seasonality	-0.16387	0.03247	27.68928	-5.047	2.51e-05 ***
Sex	0.04703	0.06864	9.67357	0.685	0.509316
Origin	-0.34784	0.06653	8.65682	-5.229	0.000616 ***

Table S6. Results from the post hoc tests on the General Activity Index Full-model.

**Type III Analysis of Variance Table with Satterthwaite's method**							**Pairwise comparison of means (emmeans)**						
	Sum Sq	MeanSq	NumDF	DenDF	F value	Pr(>F)	**Fixed Factor**	**Estimate**	**Std. Error**	**Df**	**t ratio**	**p-value**	**Cohen's *d***
Group size	0.126028	0.126028	1	28.6927	14.0624	0.0007950***	N7 – N5	0.152	0.0437	32.2	3.481	0.0015 **	0.0220
Sex ratio	0.227072	0.227072	1	28.7732	25.3371	2.358e-05***	All male – Mixed sex	0.236	0.0505	32.3	4.668	0.0001***	0.8300
Seasonality	0.228265	0.228265	1	27.6893	25.4703	2.513e-05***	Cold - Warm	0.164	0.0346	30.9	4.737	<0.0001 ***	0.7373
Sex	0.004207	0.004207	1	9.6736	0.4695	0.5093155	-	-	-	-	-	-	
Origin	0.245020	0.245020	1	8.6568	27.3399	0.0006158***	Captive - Wild	0.348	0.0853	12.1	4.076	0.0015 *	1.985

Signif. codes: '***' ≤ 0.001 '**' ≤ 0.01 '*' ≤ 0.05 '.' ≤ 0.1 ' ' ≤ 1

Table S7. Undesired Behaviors Full-model.

**Undesired Behaviors Full-model: Δ AIC = 12.29; p = 0.0004602 *****					
	Estimate	Std. Error	df	t value	Pr(>|t|)
(Intercept)	5.3552	1.8532	16.4292	2.890	0.01045 *
Group size	0.7984	0.6860	27.6144	1.164	0.25449
Sex ratio	-2.2038	0.7918	27.6599	-2.783	0.00959 **
Seasonality	-0.5166	0.5464	27.0486	-0.945	0.35279
Sex	0.7938	1.5887	7.9480	0.500	0.63086
Origin	5.5411	1.5623	7.4545	3.547	0.00846 **

Table S8. Results from the post hoc tests on the Undesired Behaviors Full-model.

Type III Analysis of Variance Table with Satterthwaite's method							**Pairwise comparison of means (emmeans)**						
	Sum Sq	Mean Sq	NumDF	DenDF	F value	Pr(>F)	**Fixed Factor**	**Estimate**	**Std. Error**	**df**	**t ratio**	**p-value**	**Cohen's *d***
Group size	3.436	3.436	1	27.6144	1.3543	0.254486	-	-	-	-	-	-	
Sex ratio	19.655	19.655	1	27.6599	7.7462	0.00959 **	All male – Mixed sex	2.2	0.846	31.1	2.606	0.0139 *	0.8421
Seasonality	2.268	2.268	1	27.0486	0.8939	0.352793	-	-	-	-	-	-	
Sex	0.633	0.633	1	7.9480	0.2497	0.630856	-	-	-	-	-	-	
Origin	31.917	31.917	1	7.4545	12.5792	0.008464 **	Captive - Wild	-5.54	2.05	12.5	-2.703	0.0186 *	-1.873

Signif. codes: '***' ≤ 0.001 '**' ≤ 0.01 '*' ≤ 0.05 '.' ≤ 0.1 ' ' ≤ 1
